# Dental chromatic alteration caused by neonatal cholestasis

**DOI:** 10.1590/S1679-45082016AI3488

**Published:** 2016

**Authors:** Yasmin Etienne Albuquerque, Camila Maria Bullio Fragelli, Josimeri Hebling, Elisa Maria Aparecida Giro

**Affiliations:** 1 Universidade Estadual Paulista “Júlio de Mesquita Filho”, Araraquara, SP, Brazil.

Cholestasis occurs due to reduced synthesis of bile acids or the deficient (intra or extra-hepatic) excretion of biliary components (cholesterol, phospholipids, bile salts, bilirubin and proteins) to the small intestine. Newborns, especially preterm-born children, have a predisposition to neonatal cholestasis because of hepatic immaturity. This condition can generate systemic problems, such as choluria, acholia, hypercholesterolemia and hyperbilirubinemia.^(1,2)^It can also change the structural composition or thickness of the mineralized dental tissues, enamel and dentine, leading to intrinsic chromatic changes due to hyperbilirubinemia (concentration of total bilirubin in blood serum higher than 5mg/dL).^(2)^ In high concentrations, bilirubin deposited into the enamel and/or dentine during the period of matrix mineralization, therefore changing its coloration permanently,^(3-7)^ since these tissues lose their metabolic activity after maturation.^(8)^

A 2-year-old girl was assisted at the pediatric clinic of the School of Dentistry at Araraquara, *Universidade Estadual Paulista “Júlio de Mesquita Filho”* (UNESP), accompanied by her father who was complaining about teeth pigmentation. The anamnesis showed that she was born in the 29^th^ week of gestation, weighing 1,800g and with an Apgar score 2 in the first minute and 4 in the fifth minute, which required urgent measures of reanimation. The patient also had hypoxia, neonatal cholestasis, persistent arterial duct (PAD), interventricular communication (IVC), anemia and sepsis. The child remained hospitalized at the intensive care unit (ICU) for 40 days, and she underwent high-intensity phototherapy along with medication to reduce the production of bilirubin. The treatment was continued until normalization of clinical condition. Tests were carried out when the child was 3 months old and the results showed total bilirubin concentration equal to 14.9mg/dL; Glutamic-oxaloacetic transaminase (GOT) of 178U/L and glutamic-pyruvic transaminase (GPT) of 165U/L. At 1 year of age, the patient’s references were within normal ranges, and she did not present any motor or late cognitive sequelae.

**Figure 1:**
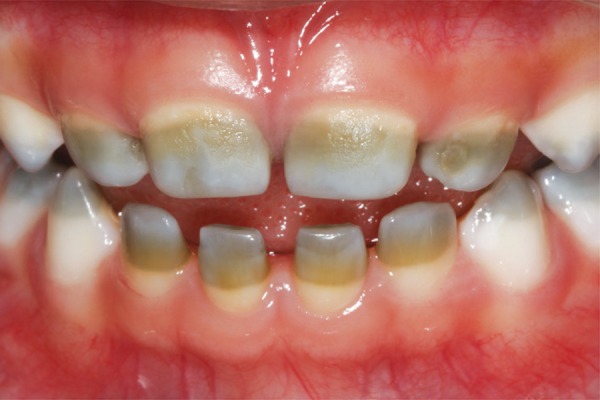
Frontal view of the dental arches showing greenish coloration in the incisal and middle thirds of deciduous incisors and canines as a consequence of neonatal cholestasis

The intraoral examination revealed the presence of a greenish colored band with a rough surface area affecting most part of the incisors crowns, and cusps of the canines and molars (Figure 1). The teeth had no carious lesions and all other evaluated aspects in the intraoral examination, such as chronology and sequence of eruption, dental morphology, gingival tissue and other soft tissues were normal.

Deciduous teeth begin their mineralization around the fourth month of intrauterine life, and incisors crowns are completed, on average, 3 months after birth. Deciduous canines and molars present only the cusps mineralized at birth and the crowns are completed between 6 and 11 months after birth.^(9)^Therefore, considering the localization of the pigmentation on deciduous incisors and canines crowns, the color change was diagnosed as a sequel of the hyperbilirubinemia and cholestasis that occurred in the child first months of life.

The follow-up is indicated in order to evaluate the impact of the disease in permanent dentition since the process of mineralization begins at birth, with the first permanent molars, and it is completed after 7 to 8 years of life (with exception of third molars).^(9)^ Therefore, there is a risk that permanent teeth will also be affected.

Reestablishment of aesthetics may be recommended for deciduous teeth, but it gains a greater importance when permanent teeth are involved. Treatment options include teeth whitening and placement of composite resin restorations.^(10)^ In the present case, the treatment plan did not prioritize aesthetical rehabilitation of deciduous teeth, but the prevention of dental caries, with application of fluoride varnish every 3 months, diet guidance and oral hygiene instruction. The follow-up has been carried out for 2 years and 11 months.

Our study sought to alert health professionals, especially dentists and dental care providers, about the importance of the medical history for the diagnosis of dental anomalies.
